# Stereotactic body radiotherapy using a hydrogel spacer for localized prostate cancer: A dosimetric comparison between tomotherapy with the newly‐developed tumor‐tracking system and cyberknife

**DOI:** 10.1002/acm2.13395

**Published:** 2021-08-20

**Authors:** Yoshihiko Manabe, Seiji Hashimoto, Hideki Mukouyama, Yuta Shibamoto

**Affiliations:** ^1^ Department of Radiology Nagoya City University Graduate School of Medical Sciences 1 Kawasumi, Mizuho‐cho, Mizuho‐ku Nagoya 467‐8601 Japan; ^2^ Department of Radiation Oncology Nanbu Tokushukai Hospital 171‐1 Hokama, Yaese‐cho Simajiri‐gun Okinawa 901‐0493 Japan; ^3^ Department of Urology Nanbu Tokushukai Hospital 171‐1 Hokama, Yaese‐cho Simajiri‐gun Okinawa 901‐0493 Japan

**Keywords:** cyberknife, prostate cancer, stereotactic radiotherapy, tomotherapy, tumor‐tracking system

## Abstract

**Purpose:**

With a new tumor‐tracking system (Synchrony®) for tomotherapy (Radixact®), the internal and set‐up margins can be tightened, like cyberknife (CyberKnife®), in the planning of stereotactic body radiotherapy (SBRT) for prostate cancer. Recently, the usefulness of placing a hydrogel spacer between the prostate and rectum has been established in prostate radiotherapy. We evaluated the characteristics of tomotherapy plans with the tumor‐tracking system and compared them with cyberknife SBRT plans for localized prostate cancer using a hydrogel spacer.

**Methods:**

In 20 patients, two plans were created and compared using tomotherapy and cyberknife. All patients underwent hydrogel spacer injection behind the prostate before simulation CT and MRI for fusion. For all plans, 36.25 Gy in 7.25‐Gy fractions for a minimum coverage dose of 95% of planning target volume (PTV) (D95%) was prescribed. The D99% of PTV and D0.1 ml of the PTV, urethra, bladder, and rectum were intended to be > 90%, 110–130%, 100–110%, <110%, and <100%, respectively, of the prescribed doses.

**Results:**

All plans using tomotherapy and cyberknife achieved the intended dose constraints. The cyberknife plans yielded better median PTV‐V110% (volume of PTV covered by 110% isodose line, 54.8%), maintaining lower median D0.1 ml of the urethra (37.5 Gy) and V80% of the bladder (11.0 ml) compared to the tomotherapy plans (39.0%; *p *< 0.0001, 38.2 Gy; *p* < 0.0001, and 18.3 ml; *p* < 0.0001, respectively). The tomotherapy plans were superior to the cyberknife plans for the rectum (V80% = 0.4 vs. 1.0 ml, *p* < 0.001; D1ml = 26.4 vs. 29.0 Gy, *p* = 0.013).

**Conclusions:**

Our results suggested that tomotherapy with the tumor‐tracking system has reasonable potential for SBRT for localized prostate cancer using a hydrogel spacer.

## 1 INTRODUCTION

Intensity‐modulated radiotherapy (IMRT) is a standard treatment for localized prostate cancer,^1^, ^2^, ^3^ but the long‐term overall treatment time remains a disadvantage, that is, 1.5‐2 months with 2.0‐2.5‐Gy daily fractions. Based on the promising results of high‐dose‐rate brachytherapy,^4^, ^5^ stereotactic body radiotherapy (SBRT) has been established for localized prostate cancer.^6^, ^7^, ^8^, ^9^ The treatment period can be shortened to 1–2 weeks using SBRT. Theoretically, since the α/β ratio of prostate cancer is presumed to be equivalent to or even lower than the ratio for late‐responding normal tissues, ultra‐hypo fractionation seems to be suitable for the treatment of prostate cancer.^10^, ^11^ Clinical evidence of SBRT for localized prostate cancer including 8‐ to 10‐year results of CyberKnife® (Accuray Inc., Sunnyvale, CA, USA) has been reported.^7^, ^8^

TomoTherapy® (Accuray Inc.) is a radiation delivery system for dynamic IMRT that is also capable of delivering stereotactic radiotherapy.^12^, ^13^ Recently, a new tumor‐tracking system (Synchrony®; Accuray Inc.) that has been available for cyberknife has been developed for clinical use in the recent tomotherapy (Radixact®; Accuray Inc.).^14^, ^15^, ^16^, ^17^, ^18^, ^19^ With this system, the internal and set‐up margins can be tightened, like cyberknife, compared to conventional tomotherapy in the planning of SBRT for localized prostate cancer.

The usefulness of placing a hydrogel spacer (SpaceOAR® System, Augmenix Inc., Waltham, MA) between the prostate and rectum has been established in prostate radiotherapy.^20^, ^21^ The purpose of this study was to verify the potential of tomotherapy with the tumor‐tracking system to generate clinically acceptable SBRT plans and compare them to cyberknife SBRT plans for localized prostate cancer using a hydrogel spacer.

## 2 MATERIALS AND METHODS

### 2.1 Study approval and patients

This study was approved by our institutional review board. The study subjects were 20 men with localized prostate cancer. All patients gave written informed consent before entry to the study. Placement of gold fiducial markers in the prostate is mandatory to implement SBRT with the tumor‐tracking system, but the patients did not undergo insertion of the markers because this was a planning comparison study, and the patients were actually treated with IMRT using tomotherapy. Patients were staged according to the 8th edition of TNM staging at clinical diagnosis and D'Amico Risk Categories,^22^ typically using computed tomography (CT), magnetic resonance imaging (MRI), and bone scintigraphy. The patient characteristics are summarized in Table 1.

**TABLE 1:** Patient and tumor characteristics

			Number
Total patients			20
Age (years)	Median (range)		77 (55‐86)
T‐stage	cT1 / T2 / T3a		6/13/1
Risk	Low / intermediate / high		4/6/10
Volume	Prostate	Median (range) (ml)	28.1 (15.9‐62.1)
	PTV		60.9 (34.6–102.4)

Abbreviation: PTV, planning target volume.

### 2.2 CT simulation and planning

**FIGURE 1:**
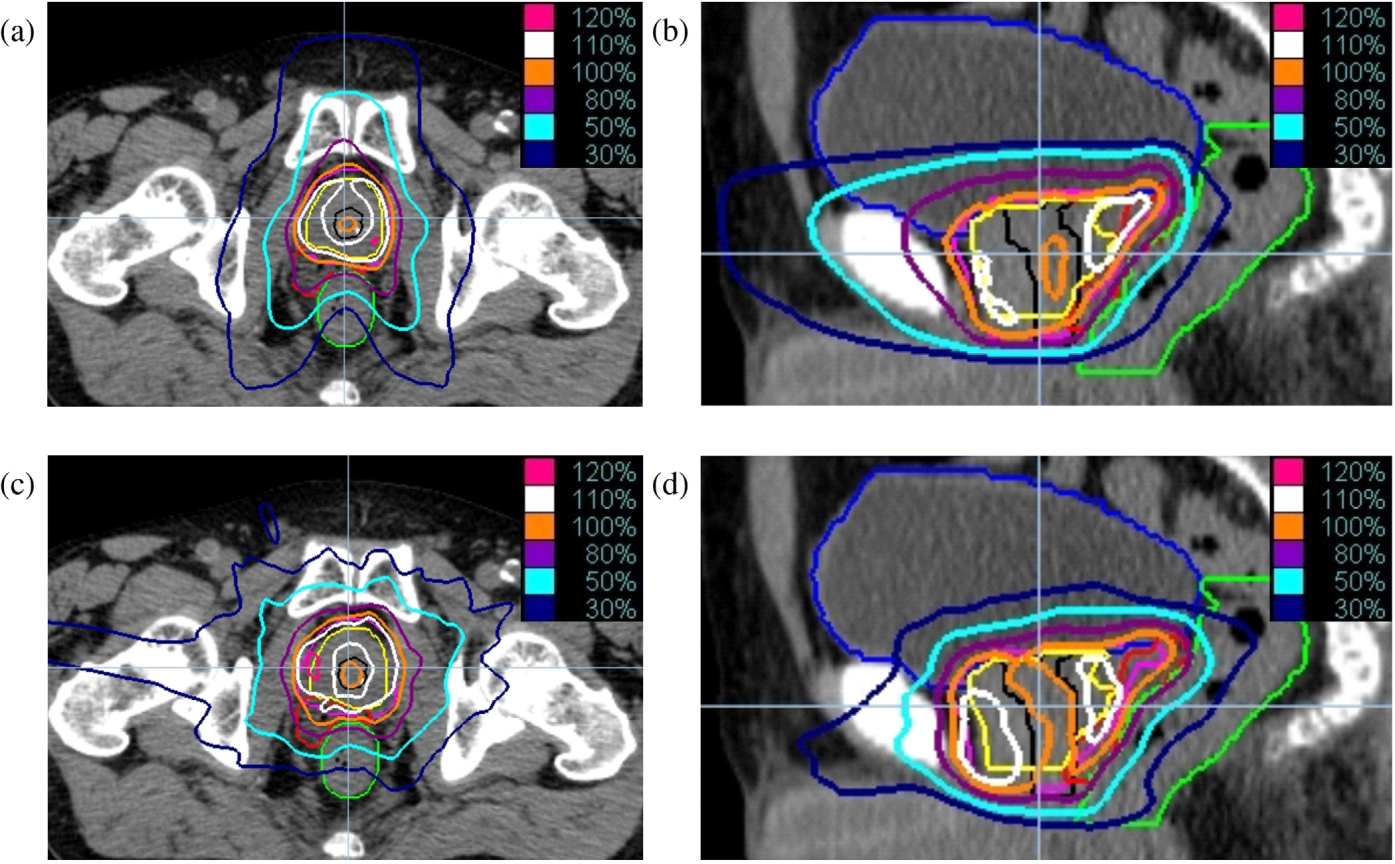
Dose distributions using tomotherapy (a and b) and cyberknife (c and d). The yellow, black, pink, red, and light‐green lines represent the prostate, urethra, planning target volume, hydrogel spacer, and rectum, respectively.

All patients underwent hydrogel spacer injection behind the prostate before simulation CT and MRI for fusion. Each patient was immobilized in a supine position with a vacuum bag system (BodyFIX; Medical Intelligence, Schwabmünchen, Germany) alongside the whole body. Axial non‐contrast‐enhanced CT and T2‐weighted MRI with a slice thickness of 2 mm were acquired for treatment planning. Contouring of target volumes and normal structures was performed on the Pinnacle^3^ version 9 treatment planning system (Philips Medical System, Eindhoven, The Netherlands). The MR images were fused to the CT images before delineation. The gross tumor volume (GTV) was defined as the prostate and proximal seminal vesicle. When the T‐stage was clinically diagnosed as cT3a, we delineated the prostate with a margin where the tumor cells spread to the capsule. The clinical target volume (CTV) was defined as GTV + 3 mm in the anterior, craniocaudal, and lateral directions and +1 mm in the posterior direction excluding the rectum and bladder. The planning target volume (PTV) was defined as CTV + 2 mm in all directions. We defined the rectum, bladder, urethra, and femoral heads as organs at risk (OARs). We delineated the urethra slightly larger than the actual urethra to deal with the prostate rotation especially along the pitch direction (Figure 1).^23^

We used some artificial structures to implement optimization smoothly: shells (PTV + 10 mm, 20 mm, and 50 mm except lateral direction [80 mm]), posterior rectum (the rectum – [PTV + 15 mm]), anterior block including the pubis, and posterior block between the rectum and skin. The contours created on the Pinnacle^3^ were exported to the other treatment planning system (Precision® version 2, Accuray Inc.) for tomotherapy and cyberknife for generation of the plans.

For all plans, 36.25 Gy in 7.25‐Gy fractions for a minimum coverage dose of 95% of PTV (D95%) was prescribed. We intended to cover a 100% (36.25 Gy)‐isodose line on the periphery of PTV and expand the area covered by a 110%‐isodose line as much as possible in the PTV. On the other hand, the maximum dose of the PTV and urethra were optimized not to exceed 130% and 110%, respectively. The dose of central area of the urethra was intended not to exceed 100%. The doses to the rectum and bladder were optimized as low as possible. We defined isodose‐line range from 30% to 120%. The dose constraints for all plans are summarized in Table 2.

**TABLE 2:** Dose constraints for all plans

Parameter		Constraints (100% = 36.25 Gy)
PTV	D99%	> 90%
	D0.1 ml	110‐130%
Urethra	D0.1 ml	< 110%
Bladder	D0.1 ml	< 110%
	D5 ml	< 105%
	D10 ml	< 100%
Rectum	D0.1 ml	< 100%
	D5 ml	< 90%
	D10 ml	< 80%
Femoral head	V40%	< 5%

Abbreviations: Dxml, minimum dose delivered to x ml of the region; D99%, minimum dose delivered to 99% of the PTV; PTV, planning target volume; V40%, percentage of the organ receiving at least 40% of the prescribed dose (36.25 Gy).

Since the first purpose of this study was to examine the feasibility of making tomotherapy SBRT plans, we did not match the dose‐volume curves for the PTV or OARs in the same patient in order to maximally utilize the abilities of each modality (tomotherapy and cyberknife). In the tomotherapy plans, a 2.5‐cm dynamic jaw was used and the pitch and maximum modulation factor were set to 0.215 and 3.0, respectively. In the cyberknife plans, multileaf collimators and the “VOLO” optimizer were used.^24^ The maximum number of nodes was set to 91. The calculation grid size was 1 × 1 mm for the final calculation process. Due to a limitation of the treatment planning system, the calculation algorithm was different for the two modalities (tomotherapy: superposition; cyberknife: finite size pencil beam). This was a planning study, so the generated plans were not implemented in practice.

### 2.3 Plan evaluation and statistical analysis

Dose distributions in the PTV and OARs and treatment times were evaluated using the paired *t*‐test with Bonferroni correction, to compare the two plans. We defined the treatment time as the beam‐on time including couch travel time for the tomotherapy plans and beam‐on and manipulator travel time for the cyberknife plans. Statistical analyses were carried out with the software package "R."^25^ Planning and evaluation were conducted by one radiation oncologist (first author).

## 3 RESULTS

Figure 1 shows a representative dose distribution in a patient. Figure 1a,b shows the dose‐distribution using tomotherapy and Figure 1c,d shows that using cyberknife for the same patient. The orange line indicates 100% (36.25 Gy), and the white line indicates 110%. In the prostate (yellow line), the area covering 110% was wider in the cyberknife plan sparing the urethra (black line) than in the tomotherapy plan. In the tomotherapy plan, the dose in the bladder spreads in the anteroposterior direction.In contrast, the rectum dose was below 80% (purple line) in almost all slices in the tomotherapy plan, while the anterior wall was covered by 80% in the cyberknife plan.

The results regarding dose distribution and treatment time of the two plans are summarized in Table 3. All plans using tomotherapy and cyberknife achieved the intended dose constraints. The cyberknife plans yielded better PTV‐V110% (volume of PTV covered by 110% isodose line), maintaining lower doses to the urethra. Bladder doses (V40%, V60%, V80%, V100%, and D0.1 ml) were lower in the cyberknife plans, whereas the tomotherapy plans were superior for the rectum (V60%, V80%, and D1ml). The treatment time was shorter in the tomotherapy plans.

**TABLE 3:** Dose‐volume parameters and treatment times of the two plans

Parameter	Unit	Tomotherapy	Cyberknife	*p* value
Median (range)				
PTV				
D90%	Gy	36.8 (36.6–37.1)	37.0 (36.7–37.2)	< 0.01
D95%		36.3 (36.2–36.3)	36.2 (36.2–36.3)	1.0
D99%		35.0 (34.2–35.5)	35.5 (35.0–35.7)	< 0.0001
D0.1 ml		45.0 (43.3–46.1)	44.5 (43.3–46.4)	1.0
V110%	%	39.0 (11.3–54.4)	54.8 (44.3–64.6)	< 0.0001
Urethra				
D0.1 ml	Gy	38.2 (37.8–39.1)	37.5 (35.9–38.0)	< 0.0001
D0.5 ml		37.9 (37.3–38.8)	37.1 (35.4–37.4)	< 0.0001
Bladder				
D0.1 ml	Gy	38.6 (38.0–39.6)	39.2 (38.2–39.8)	< 0.01
D1ml		37.5 (36.9–38.2)	37.8 (36.4–38.6)	1.0
V40%	ml	64.6 (33.8–114.2)	46.4 (16.7–78.7)	< 0.0001
V60%		38.6 (17.6–55.4)	22.9 (7.6–40.0)	< 0.0001
V80%		18.3 (8.4–25.8)	11.0 (3.8–20.5)	< 0.0001
V100%		3.8 (1.7–5.8)	2.8 (0.8–6.4)	0.023
Rectum				
D0.1 ml	Gy	31.9 (25.6–34.3)	33.1 (22.2–36.1)	0.33
D1ml		26.4 (20.4–29.4)	29.0 (16.5–32.7)	0.013
V40%	ml	10.5 (4.9–19.1)	12.9 (1.8 ‐18.4)	1.0
V60%		2.8 (0.7–5.0)	4.5 (0.1–9.1)	< 0.01
V80%		0.4 (0.0–1.1)	1.0 (0.0–3.0)	< 0.001
Time	min	8.6 (7.3–10.9)	16.0 (13.0 ‐ 20.0)	< 0.0001

Abbreviations: Dxml, minimum dose delivered to x ml of the region; Dx%, minimum dose delivered to x% of the PTV; PTV, planning target volume; Vx%, percentage or volume of the region receiving at least x% of the prescribed dose.

In the tomotherapy plans, the dose rate was 1180 monitor units/minute. The median (range) of the actual modulation factor, gantry period, and gantry rotations were 2.0 (1.8‐2.2), 35.8 (13.1‐39.1) s, and 14.2 (12.2‐35.8), respectively. The median (range) of couch traveling length and couch speed were 79.3 (66.4‐94.4) mm and 0.2 (0.1‐0.2) mm/s, respectively. In the cyberknife plans, the dose rate was 1000 monitor units/minute. The median (range) of actual numbers for nodes and segments was 38 (29‐48) and 51 (38‐71), respectively.

## 4 DISCUSSION

Since previous versions of the tomotherapy system were not equipped with a tumor‐tracking system, the intra‐fractional error was an issue in SBRT. Non‐negligible intra‐fractional error in a fraction could influence the overall treatment results because of the small fraction numbers. In addition, the beam‐on time tends to be long in SBRT to prescribe a high dose in a fraction. A study regarding intra‐fractional prostate motion showed that 95% of target positioning errors were within 2 mm using the cyberknife orthogonal tumor‐tracking system when measured every 30 s.^26^ Thus, the tumor‐tracking system allows us to tighten the margin between the CTV and PTV (PTV margin) to 2 mm. It was also reported that intra‐fractional prostate motion was not affected by a hydrogel spacer in the treatment of cyberknife.^27^ A similar tumor‐tracking system recently became available for tomotherapy systems, potentially allowing the same margin in the treatment of SBRT for localized prostate cancer using tomotherapy.

The present study revealed that the dose constraints could be fulfilled using tomotherapy. Although the hot spot area (>110% of the prescribed dose) was narrow in the tomotherapy plans, and the doses to the urethra and bladder were higher compared to cyberknife plans, the tomotherapy plans were superior regarding middle‐high dose of the rectum. It was notable that nearly maximal dose of the rectum (D0.1 ml) could be less than 90% of the prescribed dose using the hydrogel spacer. An overview of normal tissue complication probability in high dose per fraction, hypofractionated treatment effects in the clinic (HyTEC) reports showed that V_prescription dose_ < 5–10 ml for the bladder, Dmax < 35–38 Gy for the rectum, and Dmax < 38–42 Gy for the urethra were suggested when the prescription dose was 35–40 Gy in 4–5 fractions.^30^, ^31^ All plans in this study fulfilled these dose constraints (Table 3). These data suggested that tomotherapy with the tumor‐tracking system has reasonable potential for SBRT for localized prostate cancer using a hydrogel spacer.

Zelefsky et al^30^ reported that lower‐dose (32.5‐37.5 Gy in 5–6 fractions) SBRT resulted in higher rates of pathologically residual tumor at 2 years after the SBRT than higher‐dose (≥ 40 Gy in 5–6 fractions) SBRT (19%‐38% vs 11%). On the other hand, a pooled analysis of prospective phase II trials revealed no significant differences in 5‐year biochemical relapse‐free survival rates among three dose groups (35 Gy, 36.25 Gy, and 38–40 Gy in 4–5 fractions).^31^ A study regarding tumor control probability modeling for prostate SBRT showed that the probability of 5‐year biochemical relapse‐free survival rates reached the ceiling around equivalent doses of 2 Gy per fraction (EQD_2Gy_) = 90 Gy (36.25 Gy/5 fractions when the α/β ratio was estimated to 1.5 Gy) in low and intermediate risk patients. For high risk patients, the dose‐response curve seemed to still go upward from 90 Gy to 100 Gy (38.25 Gy/5 fractions) of EQD_2Gy_.^9^ However, the proportion of high risk patients of the study was relatively small (446 in 4821 patients), and the 95% confidence intervals for dose‐response were large at each point (e.g, 55–85% relapse‐free survival at 90 Gy). Thus, the importance of the hot spot area was unclear at the time of the current study.

To the best of our knowledge, evaluation of SBRT using tomotherapy with the tumor‐tracking system for localized prostate cancer has not yet been reported. A phase II study of SBRT (44‐45 Gy in eight fractions) for localized prostate cancer using tomotherapy (follow up period: 2‐25 months) showed tolerable acute toxicities (Grade 2 urinary: 23%, Grade 2 intestinal: 20%, and no Grade ≥ 3 toxicities).^13^ However, dose constraints for the prostatic urethra and the hydrogel spacer were not used, and the PTV margin was relatively large (3‐10 mm), because no tumor‐tracking system was available at the time of the study. These toxicities could be lowered using the tumor‐tracking system and dose constraints for the urethra and hydrogel spacer.

Ideally, dose‐volume curves in the PTV or some OARs should be matched in the same patient in the context of a planning comparison study. However, we did not do so to maximally utilize the abilities of each modality, since the first purpose of this study was to verify the potential of tomotherapy for clinically acceptable SBRT. Based on the results, if the V110% of the PTV in the tomotherapy plans was set to the same level in the cyberknife plans, the urethral dose will increase, and the dose constraint will not be fulfilled. As a result, such plans cannot be used in clinical practice. On the contrary, if the rectum dose in the tomotherapy plan was set to the same level in the cyberknife plans, the rectal dose restriction has to be loosened, and the ability of tomotherapy will not be brought out. In the balance of PTV coverage and OAR sparing within the dose constraints, the plans of the two systems seemed to be comparable in clinical situation.

There were a few limitations in this study. First, the slice thickness of the CT images was 2 mm because these images were obtained for IMRT in clinical practice. Thus, the dose calculation voxel size was not 1 × 1 × 1 mm but 1 × 1 × 2 mm. Second, this study is a planning study on the treatment planning system, so the characteristics of real‐time tracking for the prostate were not analyzed.

## 5 CONCLUSIONS

Our results suggested that tomotherapy with the tumor‐tracking system has reasonable potential for SBRT for localized prostate cancer using a hydrogel spacer.

## FUNDING INFORMATION

This work was supported in part by JSPS KAKENHI (grant number: JP18K15285).

## CONFLICT OF INTEREST

The authors declare that they have no conflict of interest.

## AUTHOR CONTRIBUTION

*Conceptualization, investigation, and writing–original draft*: Yoshihiko Manabe. *Resources and methodology*: Seiji Hashimoto and Hideki Mukouyama. *Writing–review and editing*: Yuta Shibamoto.

## Data Availability

The data that support the findings of this study are available from the corresponding author upon reasonable request. A part of this work was presented at the ASTRO's 62nd Annual Meeting, Miami, October 25––28, 2020.
